# Anti-NKG2D mAb: A New Treatment for Crohn’s Disease?

**DOI:** 10.3390/ijms18091997

**Published:** 2017-09-16

**Authors:** Kasper Vadstrup, Flemming Bendtsen

**Affiliations:** 1 Gastro unit, Medical Division, Hvidovre University Hospital, Kettegård Alle 30, DK-2650 Hvidovre, Denmark; kvadstru@its.jnj.com; 2 Medical Affairs, Janssen Immunology, Janssen-Cilag A/S, Bregnerødvej 133, DK-3460 Birkerød, Denmark

**Keywords:** Crohn’s Disease, IBD, NKG2D, MICA, MICB, ULBP

## Abstract

Crohn’s disease (CD) and ulcerative colitis (UC) are immunologically-mediated, debilitating conditions resulting from destructive inflammation of the gastrointestinal tract. The pathogenesis of IBD is incompletely understood, but is considered to be the result of an abnormal immune response with a wide range of cell types and proteins involved. Natural Killer Group 2D (NKG2D) is an activating receptor constitutively expressed on human Natural Killer (NK), γδ T, mucosal-associated invariant T (MAIT), CD56^+^ T, and CD8^+^ T cells. Activation of NKG2D triggers cellular proliferation, cytokine production, and target cell killing. Research into the NKG2D mechanism of action has primarily been focused on cancer and viral infections where cytotoxicity evasion is a concern. In human inflammatory bowel disease (IBD) this system is less characterized, but the ligands have been shown to be highly expressed during intestinal inflammation and the following receptor activation may contribute to tissue degeneration. A recent phase II clinical trial showed that an antibody against NKG2D induced clinical remission of CD in some patients, suggesting NKG2D and its ligands to be of importance in the pathogenesis of CD. This review will describe the receptor and its ligands in intestinal tissues and the clinical potential of blocking NKG2D in Crohn’s disease.

## 1. Introduction

Crohn’s disease (CD), an inflammatory bowel disease (IBD), is a complex immunologically-mediated chronic illness that is believed to arise due to a dysregulated immune response to microbiota in the gastrointestinal system. CD is characterized by patchy and transmural inflammation that can occur throughout the gastrointestinal tract, with alternating phases of clinical relapse and remission [1]. CD typically arises between 20 and 30 years of age with symptoms such as abdominal pain, fever, diarrhea with or without bleeding, and weight loss [2]. Despite newer and better therapies, Crohn’s disease often presents a heavy everyday burden, sometimes leading to surgery and disability [3,4]. The biologic antibodies in clinical development adopt parallel mechanisms of action with the same or other targets, namely blocking of a variety of cytokines, inhibition of the similar protein-dependent migration mechanism or neutralization of chemokines. However, other mechanisms may be involved in the pathogenesis of IBD [5,6].

The immune activating receptor Natural Killer Group 2D (NKG2D) has been implicated in the pathogenesis of IBD through its presence on intestinal cytotoxic lymphocytes and the increased expression of activating ligands on inflamed tissue [7]. Normally, the NKG2D receptor and its eight different ligands are a part of a system designed to participate in the recognition of “stressed” cells exposed to viruses or tumor growth. NKG2D is then activated on natural killer (NK) or CD8 T cells to eliminate the ligand-bearing cell as an effective defense mechanism as well as producing pro-inflammatory cytokines. For years, this pathway has been a potentially attractive approach to counter malignancies and infectious diseases by activating the system [8]. In CD, however, blocking the receptor with a biologic antibody might be a viable treatment option for lowering intestinal cell elimination and the inflammatory environment. NKG2D has been implicated in some [9,10], but not all, mouse models of colitis [11]. In human IBD, the expression and function of NKG2D have not been fully characterized [12], but a recent phase II clinical trial showed that a blocking antibody against NKG2D induced rapid clinical remission of CD in some patients, implicating NKG2D and its ligands to be of importance in the pathogenesis of CD [13].

There has been limited descriptions or a lack of focus in recent reviews of current and future treatments for IBD on this target among the many in clinical pipelines [14]. This review aims to give an overview of the expression and functional role of the NKG2D pathway in the healthy and inflamed intestine, as well as the clinical potential of a blocking antibody against the receptor in CD.

## 2. The Natural Killer Group 2D (NKG2D) Receptor

NKG2D is an activating receptor expressed on human NK, γδ T, mucosal-associated invariant T (MAIT), CD56^+^ T, and CD8^+^ T cells [8,15], which can participate in the recognition of inducible “stressed-self” ligands on the surface of target cells. This will include cells acting as intra-epithelial T cells in the intestinal wall [16]. Activation of NKG2D triggers cellular proliferation, cytokine production, and target cell killing [17]. Under normal conditions in the disease-free human system, the receptor is constitutively expressed on all the lymphocytes listed above in both peripheral blood and the intestinal tissues, but also to a low degree on a subset of CD4^+^ T cells [18].

**Figure 1:**
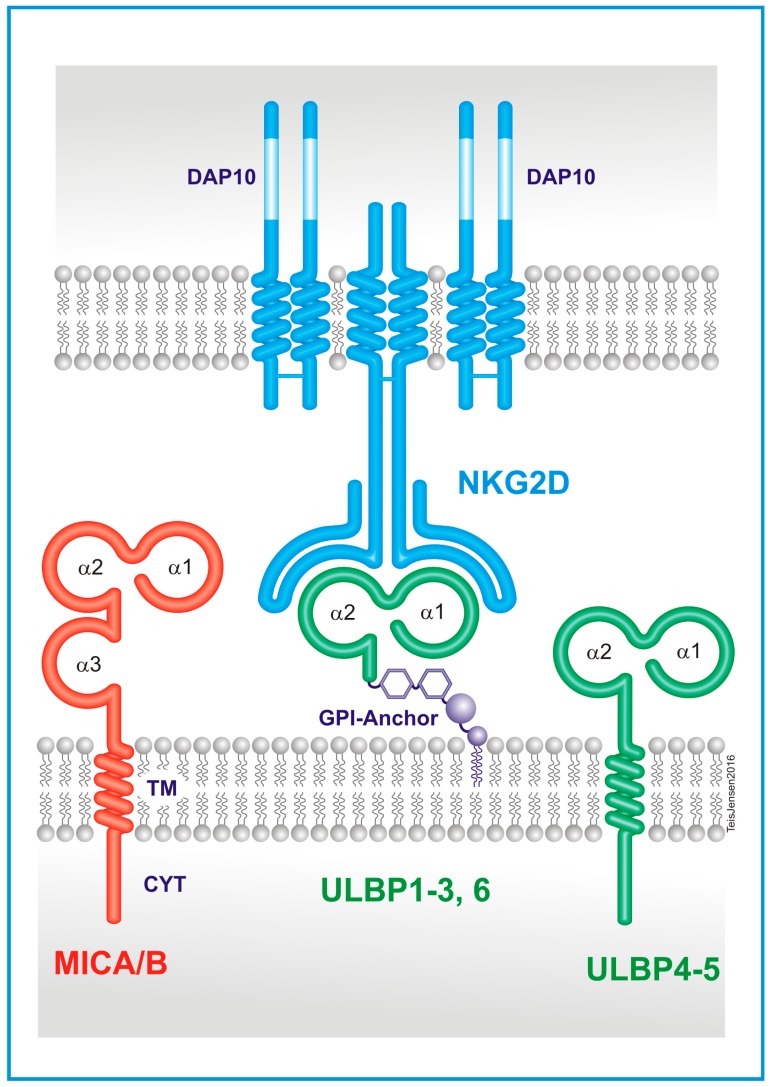
Protein structures of Natural Killer Group 2D (NKG2D) and its ligands. NKG2D is a disulfide-linked homodimer, transmembrane protein that can bind to two adapter molecules, DAP10 (DNAX-activating protein of 10 kDa), also a homodimer. NKG2D ligands are eight different MHC I-like molecules. Four of these (MHC class I polypeptide-related sequence (MIC) A/B and UL16 binding protein (ULBP) 4/5) are bound to the cell membrane by transmembrane (TM) domains with a cytoplasmic tail (CYT), while the other four (ULBP1-3 and 6) are glycophosphatidylinositol (GPI)-anchored. Each has two or three α domains.

NKG2D, or CD314, is encoded by the gene *KLRK1* (killer cell lectin-like receptor of the subfamily K member 1) and is found on human chromosome 12. The gene sits close to *KLRD1* (CD94) and the cluster of *KLRC1* (NKG2A), *KLRC2* (NKG2C), *KLRC3* (NKG2E), and *KLRC4* (NKG2F), which are both activating and inhibitory receptors binding to distinct sets of HLA-E residues [19,20]. With just two alleles different by one amino acid, the *KLRK1* gene has limited polymorphism and only one isoform encoding a functional protein has been described in humans. All mammals have *KLRK1* orthologs, indicating that the gene is highly conserved during evolution and is an important function across species [21]. NKG2D is a type II membrane protein comprising 216 amino acids with a predicted molecular weight of 25,143 kDa. The protein has an N-terminal intracellular region, a transmembrane domain, a membrane-proximal stalk region, and an extracellular region with a single C-type lectin-like domain. KLRK1 is expressed on the cell surface as a disulfide-bonded homodimer with a molecular weight of approximately 42 kDa when analyzed under reducing conditions and approximately 80 kDa under non-reducing conditions. A cysteine residue just outside the transmembrane region forms the disulfide bond joining the two subunits of the homodimer (Figure 1) [22]. In the adaptive immune system, T cells are mostly dependent on the T cell receptor complex as a result of somatic recombination of genes to recognize and respond to antigens. For NK cells in the innate system, the activation or inhibition of cell signals relies on a wide range of surface receptors where some are shared with subsets of T cells. The NKG2 receptor family are shared between cell types underlining a central role in tumor and pathogen recognition [23]. The frequency of NKG2D^+^ expression is consistently high on NK and CD8^+^ T cells and consistently low on CD4^+^ T cells in humans in the steady state, with other subsets showing differentiating expression [7,24]. While the other members of NKG2 family form heterodimers with CD94, the NKG2D receptor forms stable homodimers on the surface when stabilizing non-covalently with its adaptor molecule DAP10 (DNAX-activating protein of 10 kDa) on the inside of the cell membrane. DAP10 is also a disulfide-linked homodimer [18,25] (Figure 1). NKG2D associates with two DAP10 proteins in the transmembrane region via charged residues within the receptor and its adapter subunits and creates a hexameric complex. A charged amino acid residue (aspartic acid) centrally located within the transmembrane region of DAP10, forms a salt bridge with a charged amino acid residue (arginine) in the transmembrane region of NKG2D to stabilize the receptor complex. Upon interaction with its ligands, the complex transmits an activation signal triggering lymphocyte cytotoxic granule polarization and degranulation, cytokine production, proliferation, and survival [22].

## 3. The NKG2D Ligands

Eight human ligands for NKG2D have been identified: Human Major Histocompatibility Complex (MHC) class I polypeptide-related sequence A (MICA), MICB, and UL16 binding protein (ULBP) 1–6 (also named RAET1E, RAET1G, RAET1H, RAET1I, RAET1L, and RAET1N). All are located on chromosome 6 within the Major Histocompatibility Complex and have various binding affinities for NKG2D ranging from 10^−6^ to 10^−9^ M, with different homology between ligands down to 25%, and with extensive allelic polymorphisms [26,27]. *MICA* encodes 79 protein variants and *MICB* encodes 26 protein variants, while the *ULBP*s exhibit a lower level of polymorphism [28,29]. MICA, MICB and ULBP4 and 5 share a structure of having a transmembrane anchor in the cell and an intracellular cytoplasmic tail, while ULBP1-3 and 6 have a glycophosphatidylinositol (GPI)-anchored organization. MICA and MICB carry three extracellular immunoglobulin-like domains (α1, α2, α3,) and ULBP1–6 carry two domains (α1, α2). However, unlike class I, the ligands are not covalently bound to a monomorphic β2 microglobulin, and its peptide binding groove is empty and does not present peptides [30] (Figure 1). ULBP2 has the unique feature to be expressed at the cell surface either as a transmembrane or a GPI-anchored protein [31]. Allelic variants of the NKG2D ligands have been reported to result in large differences in the affinity of binding to NKG2D. For instance, MICA alleles with a single amino acid substitution at position 129 in the α2 domain of methionine (M) or valine (V) have been classified as having strong or weak binding affinity for NKG2D, respectively. These variable affinities have been suggested to affect thresholds of NK cell triggering and T cell modulation and consequently influencing clinical phenotypes in autoimmune disorders and malignancies [32,33,34].

NKG2D-ligand complex crystal structures have been described for some of the ligands and the binding of the different proteins seem to be adaptive fit mechanisms in the receptor [35]. Although transcripts are present in some healthy cell types [36], the ligand proteins are rarely present on the cell surface of healthy cells and tissues, but are inducible by virus infection, tumorigenesis, or by stimuli, such as DNA damage, oxidative stress, heat shock, toll-like receptor signaling, or cytokine exposure [16,37,38,39]. While not present under normal conditions, it seems that every cell type has the ability to express one or several of the ligands under one of the conditions listed above, or by the appropriate stimuli, which can be induced with pro-inflammatory signals. The induction of NKG2D ligand expression is attributed to cellular ”stress” and every type of cancer has the same mechanism. Accelerated proliferation other than cancer development can also result in upregulated ligand expression, for instance healthy cells in embryonic tissues and tissues undergoing wound repair [40,41,42]. Increased MICA expression has been reported in tissues with inflammation and autoimmune diseases such as type 1 diabetes, celiac disease, rheumatoid arthritis [43,44,45], in atherosclerosis, where it is found on vascular endothelial cells [45,46,47], and in asthma [11]. MICB has been located on many of the same sites, but the ULBPs are generally underreported in these diseases.

The regulation of NKG2D ligand transcripts is a complex process including epigenetics, RNA degradation, microRNA interference, and regulation of the protein is subject to cleavage from the surface by membrane metalloproteases or intracellular degradation by ubiquitin ligases [38]. This regulation is also different between cell types and maturation statuses.

## 4. Immunological Pathway of NKG2D

NKG2D serves as a sensor for recognition of “induced self” for the detection and removal of hyper-proliferative cells, transformed cells, or cells infected by pathogens. As with the ligands, signaling and regulation by the NKG2D receptor in NK cells and T cells is complex and incompletely understood. While NKG2D is expressed constitutively on essentially all resting human NK cells and CD8^+^ T cells [48], engagement of NKG2D alone is not sufficient to trigger cell-mediated cytotoxicity or cytokine production [49,50]. The simultaneous engagement of NKG2D and other costimulatory receptors, such as CD335 (NKp46) or CD244 can, under the right circumstances, result in cytolytic activity in resting NK cells [49]. NK cells can also be primed by culture in IL-2 or IL-15—resembling some inflammatory sites—and engagement of NKG2D alone will then be sufficient to initiate degranulation and cytokine production. The result can be increased killing of the ligand-expressing cells and/or production of cytokines, such as IFN-γ, TNF-α, and GM-CSF enhancing the inflammatory environment [51,52,53].

**Figure 2:**
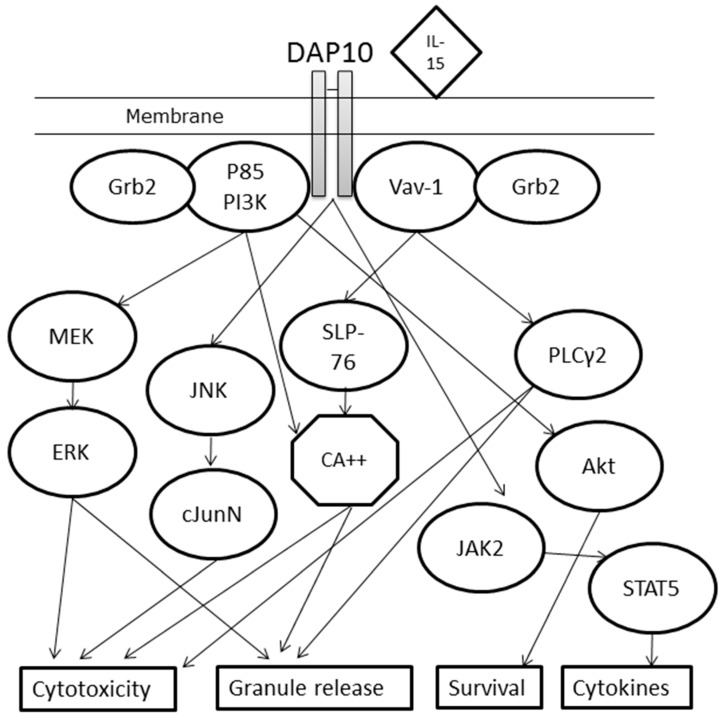
Diagram of the signaling pathways of the activated NKG2D receptor complex through DAP10. Cytokines are presented in diamond shapes, signaling proteins in ovals, ions in hexagons and cellular effects in rectangles. DAP10 (DNAX-activating protein of 10 kDa); IL-15 (interleukin 15); Grb2 (Growth factor receptor-bound protein 2); P85 PI3K (P85 subunit of PhosphoInositide 3-Kinase); Vav-1 (vav guanine nucleotide exchange factor 1); MEK (Mitogen-activated protein kinase kinase); ERK (Extracellular signal-Regulated Kinase); JNK (c-Jun N-terminal protein Kinases); cJunN (c-Jun N-terminal kinase-1); SLP-76 (SH2 domain-containing leukocyte phosphoprotein of 76kDa); CA++ (Ca^2+^ ion); PLCγ2 (Phospholipase C γ-2); Akt (Protein Kinase B); JAK2 (Janus kinase 2); STAT5 (Signal Transducer and Activator of Transcription 5).

As described, each NKG2D homodimer associates with two DAP10 homodimers to form a hexameric receptor complex (Figure 1) [25]. The related DAP12 adaptor protein was previously described as another transducer in this mechanism, but the protein, which signals through an ITAM region, does not form stable complexes with human NKG2D, unlike in mouse, thus identifying a species difference, and was reviewed in [54]. The adaptor molecule of NKG2D, DAP10 has a YINM motif, which recruits a p85 PI3 kinase and Vav-1 signaling complex [22,55]. Once DAP10 is activated, and when the complex is binding to a ligand, the stimulation leads to a PI3K-dependent Akt phosphorylation (alternatively through Vav1), which presumably activates cell survival pathways, NKG2D-mediated calcium release, cytokine production, and cytotoxicity [17]. IL-15 seems to be an essential cytokine for the phosphorylation of the YINM motif on DAP10 leading to the activation of PI3K or Vav-1 [23]. The mechanisms can be distinct, with both PI3K and Vav-1 recruiting Grb2 for downstream signaling, but Vav-1 uses the PLCγ2→Ca^2+^ or SLP-76 pathway for granule release and cytotoxicity, while PI3K signals through MEK→ERK or directly via Ca^2+^ increases. DAP10 phosphorylation can also result in cytotoxicity through JNK→cJunN. If PI3K signals Akt instead, the result seems to be increased survival of the lymphocyte. The release of pro-inflammatory cytokines is, instead, believed to happen in the DAP10→JAK2→STAT5 pathway [17] (Figure 2).

In addition to the function of being a potent activating receptor, NKG2D is reportedly involved in the normal development of NK and T cell function, and even in B cells maturation from hemapoietic precursors. These potential regulatory roles are reviewed elsewhere, but should be observed through the development of the blocking antibody against NKG2D [56].

## 5. Cancer Evasion and Infection Control

Most knowledge about NKG2D and its ligands comes from cancer and infectious disease research. NKG2D ligands are induced in cells infected with intracellular bacteria and viruses and can, for instance, be induced on dendritic cells responding to microbial pathogens [38]. Pathogens can seemingly induce expression of any of the ligands in a range of different cell types without general rules and it might be dependent on other external factors as well. However, viruses have evolved complicated mechanisms to prevent a given cell from expressing ligands or to truncate the protein to make it soluble or captured intracellularly, underlining the importance of the NKG2D receptor activation. Soluble ligand protein can then antagonize NKG2D to avoid detection by T or NK cells [57,58]. The diversity of NKG2D ligand genes and their polymorphism is likely driven by the pathogens’ evolving mechanisms to escape detection by immune cells through NKG2D pathway.

NKG2D ligands are expressed on all cell types by cancers [59]. In some cases, ligands may be induced due to the genomic instability of the transformed cells, resulting in activation of DNA repair pathways [60], but the factors causing NKG2D ligand expression depend on the transformed cells, as well as exposure to cytokines and other factors in the microenvironment [38]. Hyper-proliferation of transformed cells might be another reason for induction caused by activation of transcription factors [42]. Like viruses, primary tumors frequently develop mechanisms for avoiding detection and elimination by lymphocytes. These include systemic release of soluble NKG2D ligands [61,62]. Additionally, when NK or T cells encounter cells bearing NKG2D ligands, a mechanism down-modulates the receptor [63,64]. In addition, tumor-derived factors, such as anti-inflammatory TGF-β, can cause down-regulation of the NKG2D receptor on NK cells and T cells [65,66,67]. These pathways are further reviewed in [59].

## 6. NKG2D in Crohn’s Disease

NKG2D on lymphocytes and its ligands MICA, MICB, and ULBP1-6 modulate T and NK cell activity and may contribute to IBD pathogenesis. However, only a few studies have addressed the relation of NKG2D to IBD and especially the ligands are poorly characterized [7,12,68,69,70,71,72].

NKG2D is expressed to a high degree on NK and CD8^+^ T cells in CD also, but can also be detected on CD4^+^ T cells in inflamed CD intestine [7,73]. The expression level on CD4^+^ T cells have been shown to increase with Crohn’s disease in the lamina propria compared with controls and patients with ulcerative colitis. CD4^+^NKG2D^+^ T cells with a Th1 cytokine profile and expressing perforin were increased in the periphery and in the mucosa in CD. CD4^+^NKG2D^+^ T cell clones were functionally active through MICA-NKG2D interactions, producing interferon-γ and killing targets expressing MICA [7]. This defines a subset of CD4^+^ T cells shown to have cytotoxic and inflammatory properties. Other T cell types can show heterogeneous results between patients. The differences in the NKG2D expression with disease stage might be caused by ligand-induced internalization or due to differences in cytokines in the mucosal gut tissue, as several cytokines are known to either increase [74,75,76,77] or decrease [65,66,67,78,79] NKG2D on NK and T cell subsets. CD56^+^ αβ T and γδ T cells have previously been found to express NKG2D differently [80] and the balance between these two cell types might be a modulating factor between self-tolerance and autoimmunity [81]. Our group has found that NKG2D expression on γδ T cells (CD45^+^CD3^+^γδTCR^+^) correlate negatively with C-reactive Protein (CRP), while CD56^+^ T cells (CD45^+^CD3^+^γδTCR^−^CD56^+^) correlate positively. This might be connected to the finding that NKG2D expression on γδ T cells also correlates negatively to the cells production of GM-CSF, but positively to the release of IL-10, both measured on the protein level. The expression level of NKG2D on CD56^+^ T cells correlate to the two cytokines in the complete opposite way, which implies that pro- and anti-inflammatory cytokines influence the NKG2D molecular pathway [73]. Notably, γδ T cells constitute ~40% of the intraepithelial lymphocytes (IEL) [82,83], and might have a protective role in IBD [84]. CD56^+^ αβ T cells are activated T cells and may participate in the inflammatory response. Unlike conventional naïve T cells, IELs do not need priming and they will immediately release cytokines or mediate killing of infected target cells. CD8^+^ T and γδ T cells are most common IELs [85]. These activated T cell subsets or all available activated NK cells can release interferon-gamma (IFN-γ) and use cytotoxic killing without prior cell activation, but just by stimulation of FcRs or NK receptors such as CD94 or NKG2D by stressed or infected cells. Inhibitory lymphocyte receptors, like the heavily-studied PD-1, will ensure that self will be preserved under immunosurveillance by these innate subsets and non-self without ligands for inhibitory receptors will be killed if activated through NKG2D [86].

There could be a tendency toward the down-regulation of the NKG2D receptor on lymphocytes in inflamed CD tissue compared to non-inflamed CD tissue and normal controls. Down-regulation of NKG2D protein is not due to a decrease in NKG2D mRNA levels in CD patients versus normal controls. This lower NKG2D expression in inflamed tissue likely results from either the cytokine balance as mentioned or by increased ligand expression in the inflamed mucosal gut tissue, causing ligand-induced down-regulation of NKG2D [87,88,89].

### 6.1. Upregulated Ligands in Crohn’s Disease

Weak MICA expression has been reported on the cell surface of some healthy cell types including epithelial cells in the gut [90,91]. Increased MICA expression has also been reported in autoimmune diseases, such as type 1 diabetes, celiac disease, rheumatoid arthritis, and atherosclerosis, where it is found on vascular endothelial cells [43,44,45,46,47]. Additionally, associations between MICA alleles and thyroid disease and Addison’s disease have been observed, pointing to these factors of innate immunity contributing to the pathogenesis of autoimmune disorders [92,93]. Increased levels of MICA and/or MICB have also been observed on epithelial cells and monocytes in CD patients, where they may trigger cytokine release and cytolytic activity [7,12,68,94]. Allez et al. described how increased MICA expression could prime NKG2D^+^ CD4^+^ T cells to kill the ligand bearing cells while also producing high levels of IFN-γ and IL-15 [7]. Analyses of ULBP1-6 expression in human CD are incomplete, but ULBP1 and ULBP2 expression have been reported on intestinal monocytes from pediatric IBD patients. This study identified an increase of MICA/B^+^, ULBP1^+^, and ULBP2^+^ cells from mucosal infiltrates in tissue sections from active disease only, not when disease was in remission or in normal controls [12]. The fact that MICA is typically more expressed than MICB could be due to gene promotor polymorphisms [95], and whether this is also the case for the ULBPs needs to be addressed. mRNA differences for MICA in CD patients compared to normal controls have previously been reported [96]. Additionally, several reports have linked polymorphisms in MICA and MICB to increased prevalence of IBD [70,97,98], and other polymorphisms in MICA that, on the contrary, protects from ulcerative colitis, have been found [69].

We have recently shown the presence of all eight ligands on intestinal monocytes and B cells in CD patients by flow cytometry, as well as on endothelial and epithelial cells [73]. To be able to show this, we had to produce antibodies for ULBP3–6, which could explain the few studies on these ligands. The level of expression was highly heterogeneous and differed widely between cell types. No difference in the average expression levels was detected between MICA and MICB, and the ULBPs, but at the mRNA level, the ULBPs were generally more produced. The presence on epithelial cells could be part of the pathogenesis of CD, if lymphocytes eliminate the gut mucosal wall because of ligand presence in the inflammatory microenvironment. The expression of some of the ligands was tended to increase with higher concentrations of pro-inflammatory cytokines like IL-1β and TNF-α.

A better characterization of the expression pattern and functional role of NKG2D ligands in CD is warranted to improve the understanding of the mechanism-of-action by NKG2D blockade therapy and the pathway itself.

### 6.2. NKG2D Pathway Contribution to CD Pathology

Loss of tolerance towards microbiota crossing the epithelial barrier is likely the initiation of CD, and the pathology accelerates and persists due to the increased amount of immune cells and the mix of cytokines and chemokines creating inflammation. The pathologic response could start with the innate immune system and would then be mediated by antigen presenting cells (APCs) to activate adaptive immunity. Increased signaling or ineffective processing and clearance by epithelial cells, monocytes, neutrophils or dendritic cells (DCs) could lead to an exaggerated cytokine release resulting in an overly aggressive acquired T cell environment [99]. The inflammatory environment in turn activates other cells like cytotoxic Innate like lymphocytes (ILCs), NK cells, CD8^+^ T and CD4^+^ T cells capable of killing epithelial cells and other APCs and thus increasing inflammation. The cytotoxic cells can recognize the stress-induced ligands for activating receptors. This pathway could lead to degradation of the ligand-bearing epithelium and result in more microbial influx generating even more inflammation [100,101]. Translational research linked the properties of NKG2D-activated lymphocytes to the potential contribution to pathogenesis in Crohn’s disease. CD4^+^ T cells expressing NKG2D has been observed to be increased in CD. Like innate lymphocytes, these cells are able to kill NKG2D ligand-bearing cells and produce pro-inflammatory cytokines like IL-17, TNF-α and IFN-γ [7]. It has been shown that most of the oligoclonal expansion of mucosal T cells in CD consists of NKG2D-expressing CD4^+^ T cells [102]. This expansion will increase concentration of pathogenic TNF-α and IL-17 [103], which has been proven to contribute to disease by the registration of a biologic working through the IL-23/Th17 pathway [104,105]. The implication of CD4^+^NKG2D^+^ T cells in gut inflammation has been further shown in a murine model of transfer-induced colitis. In these studies, administration of a specific NKG2D blocking antibody decreased NKG2D expression on CD4^+^ T cells, blocked the receptor on the other lymphocyte subsets and attenuated the development of colitis, highlighting NKG2D as a possible therapeutic target in IBD [9,10]. Additionally, from coeliac disease it was shown how NKG2D modulates the disease through the cytotoxicity of NK and CD8^+^ T cells, indicating potential for a blockage in autoimmune diseases [47,106].

By blocking the NKG2D receptor with an antibody in human patients, the ligands will be physically unable to bind resulting in a reduced cytotoxic microenvironment and reduced killing of target cells in CD [107]. Our group also suggests an additional effect, namely a reduced ability of NKG2D-expressing lymphocytes to cross a ligand-expressing endothelial barrier if blocked by anti-NKG2D antibody [73]. Microvascular intestinal endothelial cells expressing MICA covered a transwell with activated human CD8^+^ T cells above. When attracted through the monolayer by a chemoattractant, blocking of NKG2D resulted in significant inhibition of T cells migrating through. In that way, fewer activated lymphocytes bearing NKG2D could enter the lamina propria of affected intestinal sites.

**Figure 3:**
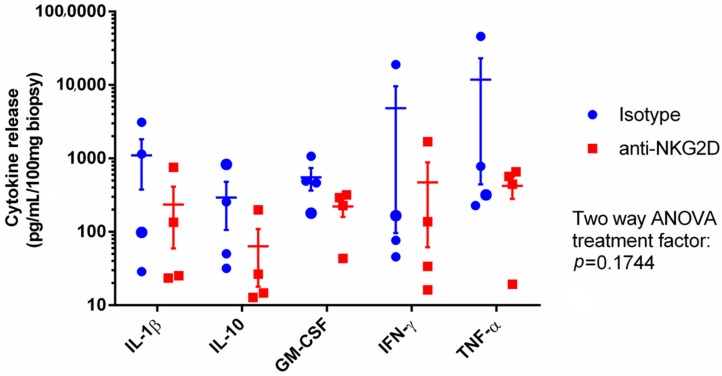
Inflamed intestinal mucosal biopsies from four CD patients treated in an explant assay over 24 h with either isotype control or anti-NKG2D antibody. The release of five cytokines to the medium was analyzed and quantified as pg/mL normalized to 100 mg of tissue. The average of measurements from four biopsies are represented in each data point, shown as the mean ± SEM, with two-way ANOVA.

Besides an increased cytotoxic microenvironment and granular release by lymphocytes activated by a NKG2D ligand interaction, the receptor can also be shown to contribute to the inflammatory balance. Using the previously described explant assay method [108], we added anti-NKG2D mAb to CD intestinal biopsies and observed a tendency towards a decreased cytokine production by the mucosal cells when the NKG2D pathway was blocked (Figure 3).

## 7. Clinical Development

### 7.1. Phase IIa Results

Animal experiments suggest a link between NKG2D and IBD, as NKG2D blockade can attenuate disease progression in certain mouse models of colitis [9,10]. In CD patients, a recent phase IIa study using a human blocking antibody against NKG2D showed significantly increased clinical remission after 12 weeks, suggesting that NKG2D-ligand interactions are viable therapeutic targets [13]. In the randomized, double-blind, parallel-group, placebo-controlled, single-dose, phase 2a trial, 78 CD patients were given either placebo or 2 mg/kg anti-NKG2D mAb and followed for 24 weeks. The primary endpoint was clinical response at four weeks. The trial was stopped prematurely primarily due to a slow recruitment rate and a negative futility analysis of the primary endpoint. However, a significant effect was achieved on the secondary endpoints at week 12 for both the Crohn’s disease activity index (CDAI) score and the Harvey Bradshaw (HBI) score (ΔCDAI = −55; *p* < 0.1 and ΔHBI = −2.7; *p* < 0.1). In patients naïve to biologic treatment, the CDAI difference from placebo was significant from week 1 through week 12 (*p* < 0.1). An exposure-response analysis for patients with CDAI > 330 was published suggesting that higher doses and repeated dosing may optimize therapeutic benefit. The failure to reach the primary end point could be due to the dose applied was too low in combination with the rather low number of patients included, but occupancy of the receptor did show to be over 80% for the first eight weeks, falling to ~20% by week 12. Higher doses have been applied in rheumatoid arthritis and a dose response study had not been performed in Crohn’s patients before initiation of the study. Furthermore, multiple dosing in the first 4–8 weeks should have been considered similar to dosage regimens for anti-TNF treatment in Crohn’s disease. The drug was well-tolerated with no evidence of immunogenicity. The findings of this small sized randomized study encourage to further randomized studies in Crohn’s disease with larger sample size, optimizing dose regimens and maybe prolongation of primary endpoint evaluation to 6–8 weeks.

### 7.2. Phase IIb Initiation

Two new clinical studies with the anti-NKG2D biologic have been initiated; a phase IIb investigating the safety and efficacy of the drug in participants with moderately to severely active Crohn's disease including 450 patients for 22 weeks with subcutaneous doses up to 400 mg induction and 200 mg every four weeks [109], and a safety study in healthy Japanese and Caucasian male participants also testing tolerability, pharmacokinetics, and pharmacodynamics following subcutaneous drug injections [110].

## 8. Future Perspectives

The introduction of anti-TNF-α monoclonal antibodies (mAb) (e.g., Infliximab) was a great advance in CD, leading to improved remission and maintenance hereof in patients with insufficient, or lack of, effect of immunomodulators [111]. The drug has low adverse effects, but only 60–70% of patients will respond and, of responders, ~40% will lose effect within the first year [112].

Targeting and blocking NKG2D would be another new mechanism of action for moderate to severe Crohn’s disease patients. Administration of a blocking antibody against NKG2D has been shown to significantly increase clinical remission in CD patients [107], most likely a result of the NKG2D blockade leading to abrogation of lymphocyte cytotoxicity and cytokine production, but it might also influence migration, recruitment, and retention of inflammatory cells into affected tissue [113]. Tissue-specific homing involves tethering, activation, and firm adhesion steps [114], and the activation might be targeted here. Activation of intestine-derived T cells has been shown to increase their migration [115]. In multiple sclerosis, a CD4^+^ T cell NKG2D-dependent migration mechanism has been observed [116]. It is therefore possible that the NKG2D and NKG2D-ligand interaction may provide an activating signal to the NK and T cells promoting successful migration, as we have proposed as a supporting mechanism [73]. In this way, anti-NKG2D antibody may uniquely interfere with both intestinal inflammation and lymphocyte homing, the two main processes targeted by current biological therapies for CD and ulcerative colitis [117,118]. Both the mechanism of cytotoxicity and the possible effect on migration should be investigated further. It would be of special interest to know which cells in the CD intestine are targeted for elimination through this pathway and which ligands play integrant roles.

More research is needed into the mechanism of action of anti-NKG2D and its therapeutic effect, especially from a genetic perspective. Single nucleotide polymorphism (SNP) is implicated in the extensive polymorphism of the ligands and relates to the binding strength of the receptor and handling of pathogens [119,120]. The single SNP at the protein level that exists for *KLRK1* can also produce differences in NKG2D function. NKG2D can vary in the intensity of cell surface expression due to genetic polymorphisms. The functional consequences of polymorphisms in NKG2D and NKG2D ligands may be cooperative or counteracting. The interaction of the variants could be highly important for the outcome of NKG2D signaling and disease associations of the NKG2D signaling pathway [119,121]. This could result in diverse efficacy between patient populations exposed to the antibody.

It could be of great interest to further investigate each NKG2D ligand to explore the exact differences between them and how they are regulated. Evolutionarily, eight ligands to one receptor indicate the importance of the interaction or differences in mechanism. The blocking of a specific ligand instead of the receptor could induce different results in CD patients.

Patients with moderate to severe Crohn’s disease, who have failed conventional treatment, and maybe some biologics, could be candidates for treatment with a drug with a new mode of action. Given the natural mechanism of NKG2D-ligand interactions in the human system, special attention should be paid to any adverse effects concerning malignancies and infections in the development and use of a blocking antibody for the use in CD.

## 9. Concluding Remarks

The NKG2D pathway poses an attractive new treatment option for Crohn’s disease. The receptor prevalence is high, its biology well-described, and it is straightforward to block the activity through a monoclonal antibody–presumably with limited adverse effects. This could potentially abrogate lymphocyte destruction of the gut tissue and the system’s contribution to the inflammatory pathogenesis. However, surprisingly little is known about the ligand prevalence and biology, especially in autoimmune diseases. Excellent commercial antibodies for all eight ligands are now available and further research should be directed towards the characterization of ligand-expressing cell types in IBD and the mechanism of action through this potent pathway.

## Abbreviations

Akt	Protein kinase B
ANOVA	Analysis of variance
CD	Crohn’s disease
cJunN	c-Jun N-terminal kinase-1
CRP	C-reactive protein
DAP10	DNAX-activating protein of 10 kDa
ERK	Extracellular signal-Regulated Kinase
Grb2	Growth factor receptor-bound protein 2
GM-CSF	Granulocyte macrophage colony-stimulating factor
GPI	Glycosylphosphatidylinositol
IBD	Inflammatory bowel disease
IEL	Intraepithelial lymphocyte
IFN	Interferon
IL	Interleukin
ILC	Innate like lymphocyte
JAK2	Janus Kinase 2
JNK	c-Jun *N*-terminal protein Kinases
mAB	Monoclonal antibody
MHC	Major histocompatibility complex
MIC	MHC class I polypeptide-related sequence
MEK	Mitogen-activated protein kinase kinase
NK	Natural killer
NKG2D	Natural killer group 2D
PLCγ2	Phospholipase C γ-2
P85 PI3K	P85 subunit of phosphoInositide 3-kinase
RNA	Ribonucleic acid
SEM	Standard error of the mean
SLP-76	SH2 domain-containing leukocyte phosphoprotein of 76 kDa
STAT5	Signal transducer and activator of transcription 5
TCR	T cell receptor
Th	T helper cell
TM	Transmembrane
TNF	Tumor necrosis factor
ULBP	UL16 binding protein
Vav-1	Vav guanine nucleotide exchange factor 1
